# Whole transcriptome analysis resulted in the identification of Chinese sprangletop (*Leptochloa chinensis*) genes involved in cyhalofop-butyl tolerance

**DOI:** 10.1186/s12864-021-07856-z

**Published:** 2021-07-09

**Authors:** Ke Chen, Yajun Peng, Liang Zhang, Long Wang, Donghai Mao, Zhenghong Zhao, Lianyang Bai, Lifeng Wang

**Affiliations:** 1 Longping Branch, Graduate School of Hunan University, Changsha, People’s Republic of China; 2 Plant Protection Institute, Hunan Academy of Agricultural Sciences, Changsha, People’s Republic of China; 3 Agricultural Biotechnology Research Institute, Hunan Academy of Agricultural Sciences, Changsha, People’s Republic of China; 4 College of Biology, State Key Laboratory of Chemo/Biosensing and Chemometrics, Hunan Province Key Laboratory of Plant Functional Genomics and Developmental Regulation, Hunan University, 410082 Changsha, People’s Republic of China; 5 Key Laboratory of Agro-Ecological Processes in Subtropical Region, Institute of Subtropical Agriculture, Chinese Academy of Sciences, 410125 Changsha, People’s Republic of China

**Keywords:** Acetyl-CoA carboxylase, Chinese sprangletop, Cyhalofop-butyl, Cytochrome P450, ATP-binding cassette transporter, RNA-Seq, Non-target site resistance, Metabolic resistance

## Abstract

**Background:**

Chinese sprangletop [*Leptochloa chinensis* (L.) Nees] is an annual malignant weed, which can often be found in paddy fields. Cyhalofop-butyl is a specialized herbicide which is utilized to control *L. chinensis*. However, in many areas, *L. chinensis* has become tolerant to this key herbicide due to its continuous long-term use.

**Results:**

In this study, we utilized a tolerant (LC18002) and a sensitive (LC17041) *L. chinensis* populations previously identified in our laboratory, which were divided into four different groups. We then employed whole transcriptome analysis to identify candidate genes which may be involved in cyhalofop-butyl tolerance. This analysis resulted in the identification of six possible candidate genes, including three cytochrome P450 genes and three ATP-binding cassette transporter genes. We then carried out a phylogenetic analysis to identify homologs of the differentially expressed cytochrome P450 genes. This phylogenetic analysis indicated that all genes have close homologs in other species, some of which have been implicated in non-target site resistance (NTSR).

**Conclusions:**

This study is the first to use whole transcriptome analysis to identify herbicide non-target resistance genes in *L. chinensis*. The differentially expressed genes represent promising targets for better understanding herbicide tolerance in *L. chinensis*. The six genes belonging to classes already associated in herbicide tolerance may play important roles in the metabolic resistance of *L. chinensis* to cyhalofop-butyl, although the exact mechanisms require further study.

**Supplementary Information:**

The online version contains supplementary material available at 10.1186/s12864-021-07856-z.

## Background

**Fig. 1:**
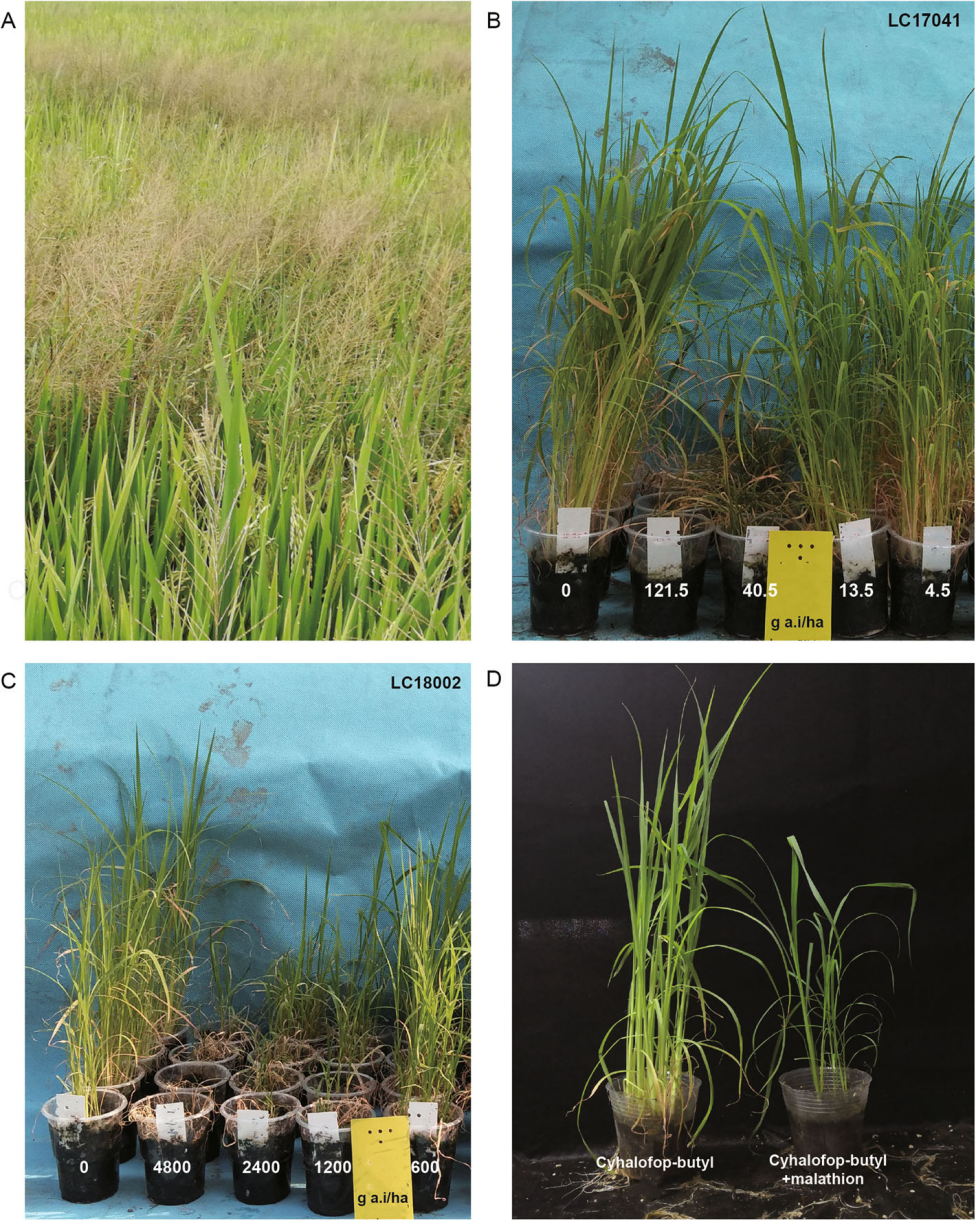
** A** The damage caused by *L. chinensis* in a paddy field. **B** Dose response of the sensitive population LC17041 to cyhalofop-butyl. **C** Dose response of the tolerant population LC18002 to cyhalofop-butyl. **D** The resistance of LC18002 to cyhalofop-butyl in the absence and presence of the cytochrome P450 inhibitor malathion

Rice (*Oryza sativa* L.) is the second largest crop in China, with a relatively stable planting area of 30 million hectare per year and an annual production of more than 210 million tons. The annual yield reduction of rice due to weed damage in China is approximately 15 %. Chinese sprangletop [*Leptochloa chinensis* (L.) Nees] is a malignant gramineous weed which causes crop loss worldwide (Fig. 1A). The damage caused by sprangletop in the rice paddy fields of China is very serious.

Cyhalofop-butyl is an acetyl-coenzyme A carboxylase (ACCase) inhibitor herbicide used in paddy fields to control *L. chinensis.* It is the most commonly utilized herbicide and possesses high activity against *L. chinensis* when deployed just after rice seedling emergence. Due to extensive and continuous use of cyhalofop-butyl, tolerance has developed in several *L. chinensis* populations [1–3]. Due to the high level of cyhalofop-butyl tolerance in recent years, it is crucial to get a better understanding of the mechanism of this tolerance in order to formulate feasible prevention and control measures.

Herbicide tolerance refers to the ability of weeds to survive a typically lethal dose of herbicide with stable heredity, rather than a temporary phenotypic response to environmental conditions [4]. The mechanisms of weed tolerance can be broadly divided into target site resistance (TSR) and non-target site resistance (NTSR) [5]. At present, research on the mechanisms of weed resistance has mainly focused on TSR. This mechanism is characterized by a change in the conformation of the herbicide target enzyme in weeds that prevents herbicide binding, or an increase in the expression of the target gene which overcomes the inhibitory effects of the herbicide [6]. In addition to TSR, other resistance mechanisms are generally classified as NTSR. NTSR is comprised of a diverse set of mechanisms and can be related to generic plant stress response or detoxification of herbicides [7].

Cytochrome P450 genes represent the largest superfamily of proteases and are involved in a host of different biological mechanisms. Hofer et al. found that the *Arabidopsis thaliana* genes CYP76C1, CYP76C2, and CYP76C4 were involved in the metabolism of benzoyl urea herbicides and played a role in the detoxification of phenylurea herbicides [8]. Saika et al. found that CYP72A31 and CYP81A6 were involved in the detoxification of bensulfuron-methyl herbicides in rice and *A. thaliana* [9–11]. In addition, cytochrome P450 enzyme activity levels have been shown to correlate with NTSR for several different tolerant weeds. During the herbicide detoxification process, detoxification products begin to accumulate in cells, eventually leading to a decrease in the activity of detoxification enzymes. Therefore, these detoxification products must be transported out of the cell in order for detoxification to continue. This is typically carried out by ATP-binding cassette (ABC) transporters. The ABC transporter family is also one of the largest and most versatile protein families found in living organisms. It has been proposed by Hart et al. that the mechanism of plant resistance to paraquat toxicity might be either the transfer of paraquat to plant cell vacuoles by ABC transporters or the enhancement of antioxidant enzyme activity [12] Yang et al. found that the NTSR of flixweed [*Descurainia sophia* L.] to tribenuron-methyl is likely the result of metabolic resistance mediated by cytochrome P450s and the movement of metabolites mediated by ABC transporters [13].

It has been reported that several populations of *L. chinensis* have developed a high level of tolerance to cyhalofop-butyl in Shanghai, Zhejiang, Jiangsu, Hunan, and other regions of China [1–3]. Despite the importance of understanding this tolerance, research has thus far mainly focused on TSR mechanisms, with very little work aimed at understanding potential NTSR.

In previous studies, we identified several populations of *L. chinensis* which were tolerant to cyhalofop-butyl, in addition to a susceptible population called LC17041. We found that when the concentration of cyhalofop-butyl reached 40.5 g/ha, the aboveground fresh weight reduction of LC17041 reached more than 80 % (Fig. 1B). In this study, we used the tolerant *L. chinensis* population LC18002 (Fig. 1C), in which application of the cytochrome P450 inhibitor malathion resulted in increased sensitivity to cyhalofop-butyl (Fig. 1D) and this population contained no mutations in ACCase, which is targeted by cyhalofop-butyl [1]. In this study, transcriptomic data from LC18002 and LC17041 were generated to identify candidate genes related to cyhalofop-butyl resistance in LC18002. This work represents the first whole transcriptomic study aimed at identifying the genes responsible for NTSR in *L. chinensis*. Identifying genes involved in NTSR is crucial to better understand the evolution of metabolic resistance, which may lead to improved weed management strategies.

## Results

### Transcriptome sequencing and screening

Sample names, standardized IDs, treatment conditions and quality control information of all samples are summarized in Additional file 1. In the standard ID, we named the tolerant population as T (tolerant), the sensitive population as S (sensitive), E (exposure) and C (control) represent herbicide exposure and control, respectively. This naming convention resulted in four groups, termed, SE, SC, TE and TC. RNA obtained from the different *L. chinensis* samples was paired-end sequenced using an Illumina NovaSeq 6000. The raw reads were then trimmed to remove adaptors, repetitive sequences and low-quality reads. The average GC content was approximately 53 %, Q20 value was above 96 %, Q30 value was above 89 %, and the Q30 value of 95 % of all samples was above 91 %. Clean reads were then mapped against the reference genome using Hisat2 [14]. Mapping rate, number of clean reads, clean bases, and additional statistics are also shown in Additional file 1.

**Fig. 2:**
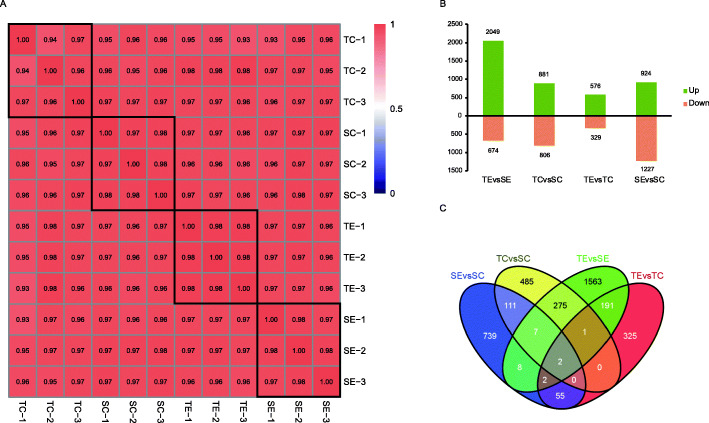
** A** Correlation pattern of each sample. Pearson correlation coefficients of samples within and between groups were calculated with the TPM of all genes in each sample. The black box represents the correlation of three replicates of the same sample. **B** Statistics on the number of DEGs in the four comparison groups. **C** Venn diagram of DEGs in different treatments of *L. chinensis* “LC18002” and “LC17041”. TE is the herbicide treated group of tolerant population, and SE is the herbicide treated group of sensitive population. TC is the control group of tolerant population, and SC is the control group of sensitive population

We next employed correlation analysis for the TPM values of each sample to determine whether replicates had similar expression values, which is a well-established method for assessing data quality. Our analysis revealed that the majority of replicates had correlation coefficients of 0.97 or higher (Fig. 2A).

### Identification of transcription factors and differential gene expression

Transcription factors (TFs) are a group of proteins that can bind to a specific sequence upstream of a gene, to ensure the expression of the target gene at a specific time and space with a specific intensity. Transcription factor prediction was analyzed by iTAK [15] software and HMMSCAN [16] was used to identify TFs. Transcription factors were compared to the plant specific transcription factor database PlnTFDB [17]. A total of 1353 transcription factors from the transcriptome were identified (Additional file 9). TFs form complex regulatory networks that control the expression of their target genes. These regulatory circuits are pivotal for coordinating transcriptional and post-transcriptional control of target genes [18]. For example, a negative regulator of DELLA genes has been shown to cause dwarfism in oil plants [19]. In *Prunus*, TFs control many agriculturally important traits such as the flowering, fruit quality, and biotic and abiotic stress resistance [20].

DESeq2 software [21] was then used to analyze the differential expression of genes in each sample, and the corrected p-value and Padj value of the differential expression tests were calculated [21]. The input data for differential expression analysis consisted of read counts obtained via quantitative analysis (Additional file 2).

As shown in Fig. 2B, there were 2,723 differentially expressed genes (DEGs) in TE versus SE, among which 2,049 genes were induced and 674 genes were suppressed. There were 1,687 DEGs in TC versus SC, among which 881 genes were induced and 806 genes were suppressed. There were a total of 905 DEGs in TE versus TC, among which 576 genes were induced and 329 genes were suppressed. There were 2,151 DEGs in SE versus SC, among which 924 genes were induced and 1227 genes were suppressed.

In order to further screen candidate herbicide resistance genes, we believe that the TC versus SC was used to compare the difference between the tolerant and sensitive material, while TE versus SE was used to compare the different results of cyhalofop-butyl treatment in the two *L. chinensis* populations. SE versus SC was used to measure the gene expression changes in sensitive *L. chinensis* genes caused by cyhalofop-butyl treatment. TE versus TC was used to determine which genes were induced by cyhalofop-butyl treatment in tolerant *L. chinensis* genes. The four sets of induced-DEGs were plotted on a Venn diagram (Fig. 2C). A total of 485 DEGs were found exclusively in the TC versus SC group, indicating that the two materials were different even without cyhalofop-butyl treatment. A total of 1563 DEGs were found uniquely in the TE versus SE comparison, meaning these genes were only different during treatment with cyhalofop-butyl. Additionally, 285 genes were differentially expressed in this set as well as the B1 versus A1 comparison (Fig. 2C), indicating that these genes were differentially expressed under both normal conditions and cyhalofop-butyl treatment. According to previous studies, resistance genes are highly expressed in resistant populations after being induced by herbicides [22, 23]. We believe that DEGs in TE versus SE therefore represent possible candidates for the NTSR present in the tolerant *L. chinensis* line. Another 739 genes were found to be differentially expressed only in the SE versus SC comparison group, indicating that they represent a response present only in *L. chinensis* that is sensitive to cyhalofop-butyl. In addition, 325 DEGs existed exclusively in the TE versus TC comparison, indicating that they represent a response present only in *L. chinensis* that is resistant to cyhalofop-butyl.

### Functional annotation of DEGs and selected candidate genes

We primarily focused on genes which were differently expressed between cyhalofop-butyl treated sensitive and tolerant plants, which may represent targets for understanding the NTSR mechanism [22, 23]. GO and KEGG enrichment analysis were performed on the differentially expressed genes in the following groups. Among them, we focused on the cytochrome P450 gene family and the ABC transporter gene family because cytochrome P450 genes have previously been implicated in herbicide metabolism [24], while CYP76 family genes are involved in the metabolism of benzoyl urea herbicides and have been shown to play a detoxification role for benzoyl urea herbicides [8]. Researchers have found that CYP72A31 and CYP81A6 are involved in the detoxification of bensulfuron-methyl herbicides in rice and *A. thaliana* [9–11]. However, no studies on the role of cytochrome P450 family genes in the regulation of cyhalofop-butyl tolerance have been conducted in *L. chinensis* using whole transcriptomic methods. In higher plants, ABC transporters have been implicated in detoxification of xenobiotics, including herbicides. Unlike cytochrome P450, which detoxifies herbicides through metabolic mechanisms, ABC transporters detoxify herbicides and confer herbicide tolerance by transporting herbicides and the metabolites that result from their breakdown [25].

### Expression analysis of tolerant versus sensitive genotypes without herbicide exposure: TC versus SC

**Fig. 3:**
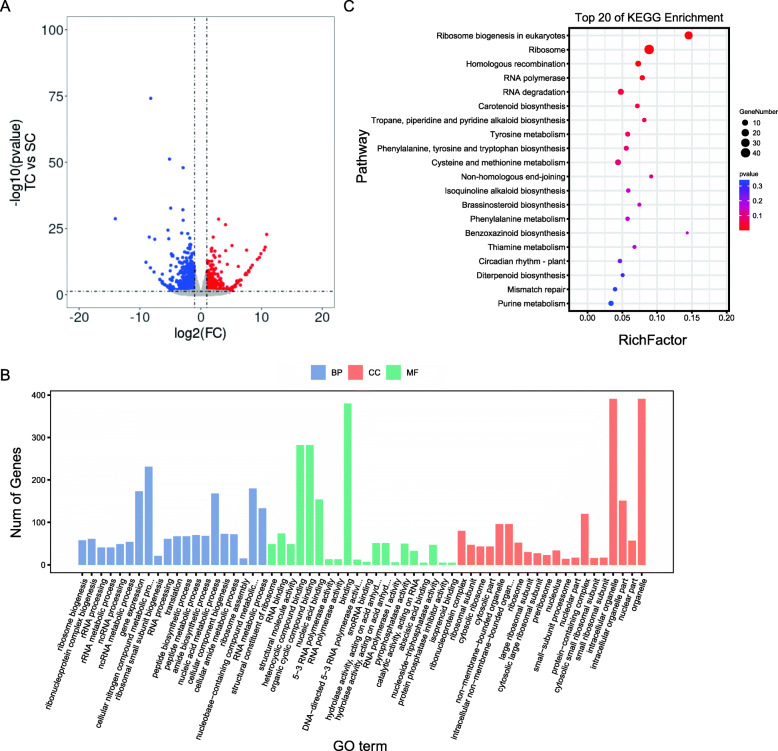
** A** The volcano plot of differentially expressed genes in TC versus SC; **B** Gene ontology (GO) analysis of induced-DEGs in TC versus SC. The DEGs were summarized in biological process, cellular component and molecular function. **C** KEGG annotation of induced-DEGs in TC versus SC. The Rich factor is the ratio of the number of DEGs annotated in a pathway term to the total number of genes in that pathway

A total of 1687 DEGs were identified in this group, among which 881 genes were induced and 806 were suppressed (Fig. 3A). The induced genes were primarily associated with biological processes such as cellular nitrogen compound metabolic, nucleic acid metabolic process, and nucleobase − containing compound metabolic. These genes were also enriched in molecular functions, such as binding, heterocyclic compound binding, and organic cyclic compound binding and enriched in cell components such as intracellular organelle and organelle (Fig. 3B). KEGG pathway analysis showed that some genes associated with ribosome biogenesis in eukaryotes and ribosome pathways were highly expressed in tolerant *L. chinensis* (Fig. 3C). Analysis of all induced genes showed that there were 10 ABC transporter family genes with higher expression in the tolerant population. Interestingly, we found an *ABCC8*(*Chr13.g36597* whose log2foldchange is 1.12) gene. It has recently been identified as involved in regulating glyphosate resistance [25]. We believe that it can transport not only the harmful components of glyphosate but also the harmful components of cyhalofop-butyl. Additionally, 14 CYP450 family genes were identified to be highly expressed in the tolerant population. A CYP71A family genes (*Chr1.g01584*, whose log2foldchange between TC and SC was 1.53) were identified in this subset, which were highly expressed in tolerant population. Interestingly, this gene is also differentially expressed in TE versus SE. Members of the CYP71A family have been identified as herbicide metabolic resistance genes [23]. Therefore, we hypothesized that these genes might be impacting the NTSR of LC18002.

### Expression analysis of tolerant versus sensitive genotypes with herbicide exposure: TE versus SE

**Fig. 4:**
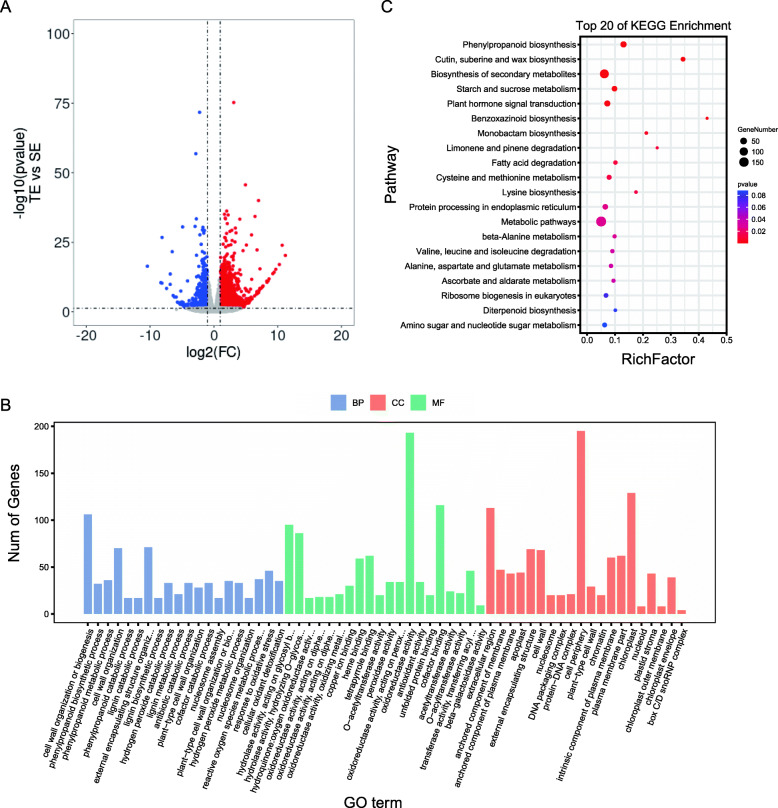
** A** The volcano plot of differentially expressed genes in TE versus SE. **B** Gene ontology (GO) analysis of induced-DEGs in TE versus SE. The DEGs were summarized in biological process, cellular component and molecular function. **C** KEGG annotation of induced-DEGs in TE versus SE. The Rich factor is the ratio of the number of DEGs annotated in a pathway term to the total number of genes in that pathway

In this group, 2,723 DEGs were identified, among which 2049 genes were induced and 674 genes were suppressed (Fig. 4A). A total of 1563 DEGs only existed in this subset. These genes showed no difference in expression when the two materials were not treated but showed differences in expression during cyhalofop-butyl treatment. Additionally, 285 genes were differentially expressed in this set as well as the TC versus SC comparison (Fig. 2C), indicating that these genes were differentially expressed under both normal conditions and cyhalofop-butyl treatment. GO term analysis of biological processes resulted in the identification of induced genes related to hydrogen peroxide metabolic process, cell wall organization or biogenesis, and reactive oxygen species metabolism. These genes were also enriched in molecular functions, such as oxidoreductase activity, hydrolase activity, hydrolyzing O − glycosyl compounds, ABCC15 gene (Chr4.g11866) were identified in the TE versus TC comparison and enriched in cell components such as cell periphery, chloroplast and extracellular region (Fig. 4B). KEGG pathway analysis results indicated that a total of 175 induced genes were identified as being involved in different metabolic pathways. One hundred and twenty-six were involved in secondary metabolite biosynthesis pathways (Fig. 4C). These findings further support our hypothesis that metabolic genes and transport genes mediate the high cyhalofop-butyl tolerance in the LC18002 line. Additionally, 8 ABC transporter family genes were identified among the induced-DEGs, 5 of which were differentially expressed only in this subset. Among the induced genes, we found that *ABCB2* (*Chr10.g32568*), whose log2foldchange between TE and SE was 2.83 were highly induced. In addition, we found that the expression of *ABCC15(Chr4.g11866)* was up-regulated not only in this set, but also in TC versus SC. This finding indicates that these genes represent candidate for further study of cyhalofop-butyl tolerance in LC18002. Cytochrome P450 family genes have been shown to play a crucial role in herbicide metabolism [24], and we found 16 cytochrome P450 family genes which had higher expression levels in LC18002. In particular, we found that a CYP71A1 gene (*Chr1.g01584*) whose log2foldChange between TE and SE was 1.32 and which not only in this comparison, but also in the TC versus SC comparison. In addition, we found that an CYP71A1(Chr4.g10580) whose log2foldchange between TE and SE was 1.60 and a CYP76(Chr1.g02164) whose log2foldchange between TE and SE was 1.39 showed no difference in TC versus SC, but were induced in this set. It has previously been found that CYP71s and CYP76s play a role for herbicides tolerance [8, 23]. Given that other members of the cytochrome P450 family have been implicated in herbicide tolerance, higher expression of these genes may play a role in the tolerance of LC18002 to herbicides. Additionally, we found 10 possible xyloglucan endo-transglucosylase/hydrolase encoding genes, and other genes of this class have previously been reported to participate in abiotic stress in pepper [26] and tea [27]. In addition to the above genes, some other key genes involved in herbicide tolerance pathways were also found in this subset. Pectate lyases have been shown to play an important role in herbicide tolerance by regulating the composition of polysaccharides in plants to affect plant stress tolerance [28]. A pectate lyase genes were identified in this subset, with log2foldchanges of 1.07 and high expression in LC18002. Six aquaporin nipgenes were also found to be highly expressed in LC18002, and members of this class are known to control pore size with significant impacts on herbicide tolerance. Peroxidase genes have also been shown to play a part in abiotic stress tolerance and herbicide tolerance by regulating the jasmonic acid biosynthesis pathway [29, 30].

### Effects of herbicide exposure on the sensitive genotype: SE versus SC

A total of 2,151 DEGs were identified in this group, among which 924 genes were induced and 1227 genes were suppressed after herbicide treatment (Additional file 7A). This group can be used to investigate which genes were differentially expressed following cyhalofop-butyl treatment in the sensitive population. GO term analysis of biological processes indicated that many induced genes were related to phosphorus metabolic process, and phosphate containing compound metabolic process. These genes were also enriched in molecular functions, such as small molecule binding, nucleotide binding and nucleoside phosphate binding and enriched in cell components such as membrane, intrinsic component of membrane and integral component of membrane. (Additional file 7B). KEGG pathway analysis showed that thirteen genes with higher expression after cyhalofop-butyl treatment were involved in plant hormone signal transduction (Additional file 7C) Six genes were involved in glycerophospholipid metabolism process, while five genes were involved in fatty acid degradation such as alcohol dehydrogenase and amino acid permease. Thirteen ABC transporter family genes with differential expression were identified in this group. Among them, twelve were induced and one was repressed. The induction of these different genes in the sensitive population likely represents a response to stress caused by cyhalofop-butyl treatment, including ABC transporters being used to transport active herbicide molecules.

### Effects of herbicide exposure on the tolerant genotype: TE versus TC

A total of 905 DEGs were identified in this group, among which 576 genes were induced and 329 genes were suppressed (Additional file 8A). This group can be used to investigate gene expression changes caused by cyhalofop-butyl treatment in the tolerant line LC18002. GO term analysis of biological processes indicated that many induced genes were related to carbohydrate metabolic process, catabolic process. These genes were also enriched in molecular functions, such as oxdoreductase activity and enriched in cell components such as membrane, integral component of membrane, intrinsic component of membrane and membrane part (Additional file 8B). KEGG analysis showed that 55 induced genes were annotated as being involved in metabolic pathways and 34 genes were involved in the biosynthesis of secondary metabolites (Additional file 8C). Six ABC transporter genes were found to be herbicide-induced in this subset. Interestingly, we found an ABCC8 gene that has recently been found to be involved in the regulation of herbicide resistance [25]. Additionally, 13 cytochrome P450 genes were highly expressed in the herbicide-exposed tolerant line. The expression levels of these genes may relatively constant between the two materials prior to treatment but their expression was significantly induced in the tolerant line after treatment with cyhalofop-butyl. It therefore is likely that these genes may play some roles in the herbicide tolerance of LC18002, although further research is necessary to confirm this.

### Phylogenetic analysis of candidate genes

**Fig. 5:**
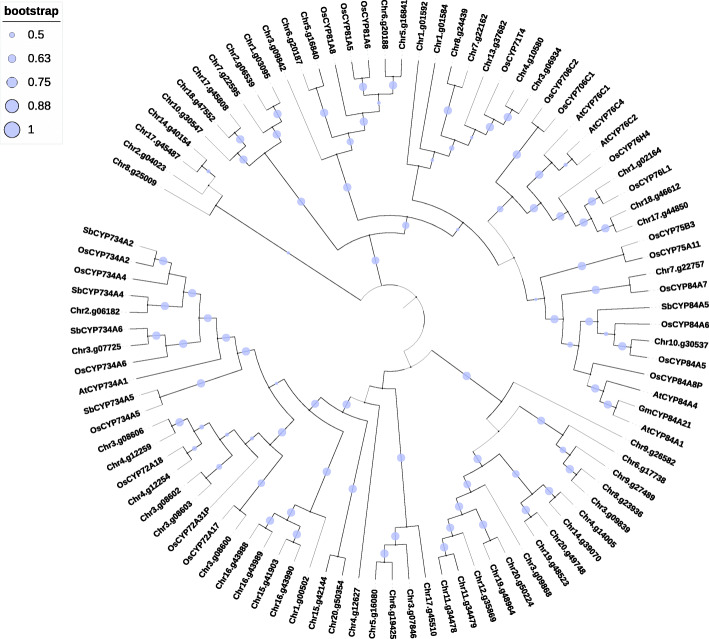
Phylogenetic tree of some candidate genes and homologous genes. The neighbor-joining tree was generated using the protein sequences of all differentially expressed cytochrome P450 family genes and cytochrome P450 genes from A. thaliana, rice, soybean, sorghum, and maize

Phylogenetic analysis was performed to determine whether the candidate genes identified had homologs in other species or belonged to any known gene families. The protein sequences of all differentially expressed cytochrome P450 family genes were compared against cytochrome P450 genes from *A. thaliana*, rice, soybean, sorghum, and maize (Additional file 13). We constructed a phylogenetic tree after multi-sequence alignment based on the above protein sequences using ClustalW [31] (Additional file 11).This clustering analysis revealed that *L. chinensis* cytochrome P450 genes clustered closely with cytochrome P450 genes of other species (Fig. 5). Additionally, we also included herbicide metabolism genes previously studied, such as *OsCYP81A6* and *OsCYP72A31P*. This analysis revealed five genes that were closely related to *OsCYP81A6*, and seven genes that were closely related to *OsCYP72A31P*.

### qRT-PCR validation of RNA-seq expression patterns

**Fig. 6:**
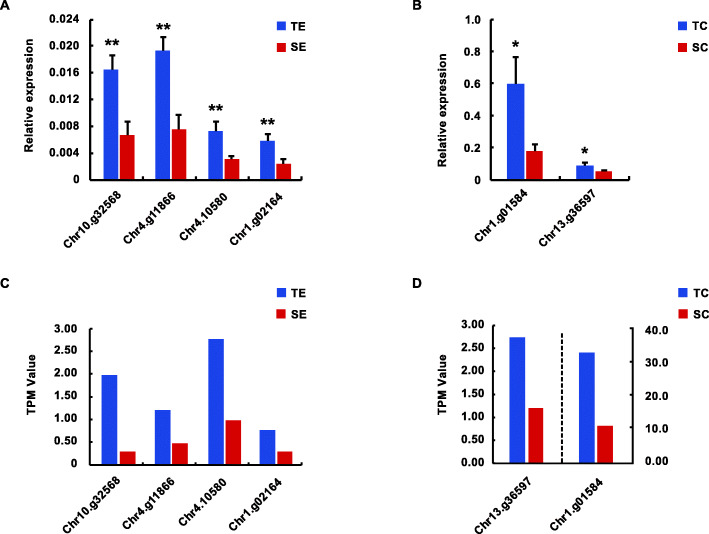
Verification of candidate genes’s expression between cyhalofop-butyl susceptible (LC17041) and resistant (LC18002) *L. chinensis* populations by qPCR. **A** Verification of the candidate genes selected in TE versus SE. The abscissa is the gene ID, and the ordinate is the relative expression values of the genes. TE is the herbicide treated group of tolerant population, and SE is the herbicide treated group of sensitive population. **B** Verification of the candidate genes selected in TC versus SC. The abscissa is the gene ID, and the ordinate is the relative expression values of the genes. TC is the control group of tolerant population, and SC is the control group of sensitive population. For all expression analyses, actin and 18 s RNA was used as reference. Error bars indicate mean + SD of biological triplicates (*P-value, t-test < 0.05; **P-value, t-test < 0.01). **C** TPM values of candidate genes between TE and SE. **D** TPM values of candidate genes between TC and SC. The genes to the left of the dotted line use the left Y-axis, and the genes to the right use the right Y-axis

In order to confirm the magnitude of candidate genes expression obtained by the transcriptomic approach (Additional file 10), the expression levels of 6 candidate genes were measured in two *L. chinensis* accessions using quantitative real-time PCR (qRT-PCR) (Fig. 6). The primers utilized to amplify candidate genes were designed based on the sequence of the identified genes and are listed in Additional file 12. The results indicated that the RNA-seq data and qRT-PCR data mostly agreed.

## Discussion

Metabolism-based resistance to ACCase inhibitor herbicides has long been reported and is increasing in weed species [32]. However, there are few studies on herbicide metabolism and resistance genes at the whole transcriptome level, which has slowed the development of new control mechanisms. RNA-seq has been successfully used to identify genes involved in metabolic resistance to ALS herbicides in two grass weed species: *L. rigidum* [33] and *A. myosuroides* [34]. However, there has been no study focused on the NTSR mechanisms of *L. chinensis* at the whole transcriptome level. Identifying genes involved in NTSR is important for understanding the evolution of metabolic resistance and improving weed management strategies in the field.

In this study, 266,750,156 clean reads were generated from *L. chinensis* by Illumina NovaSeq 6000 technology. After identifying DEGs, we found that the number of differentially expressed genes in TE VS TC was not particularly high, which may be due to the low dose of herbicide applied in the sequencing samples. Interestingly, a similar pattern has been found in other studies [35]. In the selection of candidate genes for herbicide resistance. We primarily focused on genes which were differentially expressed between sensitive and tolerant plants after being treated with herbicide. In this study, we show that tolerance to cyhalofop-butyl in LC18002 is likely due to NTSR mechanisms. Two CYP71A family genes (Chr4.g10580, Chr1.g01584), a CYP76 family genes (Chr1.g02164), a ABCB2 gene (Chr10.g32568) and a ABCC15 gene (Chr4.g11866) were identified in the TE versus TC comparison. Additionally, a CYP71A family gene (Chr1.g01584) were differentially expressed not only in TE versus SE but also TC versus SC, implying that these genes had different expression patterns both with and without cyhalofop-butyl treatment. In addition, we have also identified an ABCC8 gene (*Chr13.g36597*) differentially expressed in TC versus SC, which has recently been identified as involved in regulating herbicide resistance [25]. The chromosomal positions, GO enrichment, KEGG enrichment, Readcounts and TPM value in each sample and other information of candidate genes is included in Additional file 10. In addition, we have identified some xyloglucan endo-transglucosylase/hydrolase encoding genes,pectate lyases genes, aquaporin nip genes in DEGs, which have been identified as being involved in the regulation of herbicide resistance or abiotic stress. It is hoped to provide theoretical guidance for further research.

The CYP450 family of genes are known to play a significant role in herbicide metabolism [24]. We constructed a phylogenetic tree of all differentially expressed cytochrome P450 family genes in *L. chinensis* and some cytochrome P450 genes with herbicide metabolism function that have been identified in other species, which could be utilized for further studies on *L. chinensis* herbicide resistance and the cytochrome P450 family.

## Conclusions

This is the first report utilizing whole transcriptome analysis to identify putative herbicide tolerance genes in *L. chinensis*. An *L. chinensis* population highly resistant to the ACCase inhibitor herbicide cyhalofop-butyl, which also lacks any mutations in the target proteins for this herbicide, was selected for this analysis. The NTSR observed for this line is likely due to cytochrome P450-mediated metabolic resistance and ABC transporter mediated sequestration of the metabolites. *CYP76M5* (*Chr1.g02164*), two CYP71A family genes (*Chr4.g10580*and *Chr1.g01584*), ABCB2 (*Chr10.g32568*), ABCC15 (Chr4.g11866) and *ABCC8* (*Chr13.g36597*) were all differentially expressed in tolerant compared to susceptible *L. chinensis* lines, making them possible markers or new target genes (Additional file 10). In addition, we identified several P450 family members in the DEGs, some of which were revealed by phylogenetic analysis to be similar to known herbicide tolerance genes. We found several other DEGs belonging to families associated with herbicide tolerance, including ABCs, DELLA genes, xyloglucan endotransglucosylase/hydrolases, glutamate dehydrogenases, methyl crotonoyl carboxylases, aquaporin genes, and thaumatins, all of which represent targets for additional investigations. Overall, this study represents a significant step forward in understanding NTSR in *L. chinensis* and provides a number of new avenues to explore in future studies.

## Methods

### Plant material

*L. chinensis* seeds with two contrasting genotypes of herbicide tolerance (LC18002) and herbicide susceptibility (LC17041) were ground by grinding paper for 30 s. The seeds were soaked in water for 8–24 h, then washed and air-dried. Next, the seeds were poured into a 10.5 cm diameter plastic cup through a 20-mesh sieve, and water was added to moisten the soil completely. The seeds were then spread evenly over the moist soil and a thin layer of soil was placed on top. After sowing, the seeds were cultured in an artificial climate chamber. Two different growth conditions were utilized. The first stage was 27.0℃, 0 Lx light, and 10 h. The second stage was 32.0℃, 20,000 Lx light, 14 h.

### Cyhalofop-butyl dose response in the absence and presence of the cytochrome P450 inhibitor malathion

Once the plants matured to the 4 ~ 5-leaf stage, whole-plant response experiments were conducted to determine the sensitivities of LC18002 and LC17041 populations to cyhalofop-butyl in the absence and presence of malathion. Malathion at the rate of 720 g A.I. / HA had no visual effects on *L. chinensis* seedling growth, and was therefore used to treat the plants 30 min prior to cyhalofop-butyl treatment. Cyhalofop-butyl 1 g A.I. / HA (the dose causing slight dwarfing of sensitive population LC17041 and no yellow leaves) was sprayed with water for control. The spray device was the 3WP-2000 walking spray tower produced by Nanjing Institute of Agricultural Mechanization Research, Ministry of Agriculture. The sprinkler head was TP65015E, the flow rate was 590 mL/min, the walking speed was 482 mm/s, and the spray pressure was 0.3mpa. A total of 100 mg of young leaf tissue were harvested after 24 h of treatment and each treatment included three replicates. After harvesting, the leaves were immediately frozen in liquid nitrogen at − 80 °C prior to total RNA isolation.

### RNA extraction and quality determination for RNA-Seq

Total RNA was extracted from leaves of two individual tolerant and sensitive plants. Each population had three treatments, and each treatment included three replicates. The RNA of *L. chinensis* was extracted with the TransGen RNA extraction kit (TranGen, Beijing, China). RNA degradation and contamination were monitored on 1 % agarose gels. RNA purity was checked using the NanoPhotometer® spectrum photometer(IMPLEN, CA, USA), and RNA concentration was determined using the Qubit® RNA Assay Kit (Qubit®2.0Flurometer, Life Technologies, CA, USA). RNA integrity was assessed using the RNA Nano 6000 Assay Kit (Agilent Bioanalyzer 2100 system, Agilent Technologies, USA). The best quality RNA samples were chosen for cDNA library preparation.

### Library preparation, and transcriptome sequencing

A total amount of 3 µg RNA per sample was used as input material for the RNA sample preparations. Sequencing libraries were generated using the NEBNext® Ultra™ RNA Library Prep Kit for Illumina® (NEB, USA) following manufacturer’s recommendations. The mRNA was purified from total RNA using poly-T oligo-linked magnetic beads. Fragmentation was carried out using divalent cations under elevated temperature in NEB Next first strand synthesis reaction buffer (5X). First strand cDNA was synthesized using random hexamer primer and M-MuLV reverse transcriptase (RNaseH). Second strand cDNA synthesis was subsequently performed using DNA polymerase I and RNase H. The remaining overhangs were converted into blunt ends via exonuclease/polymerase activities. After a denylation of 3’ ends of DNA fragments, NEBNext adaptors with hairpin loop structure were ligated to prepare for hybridization. In order to select cDNA fragments of preferentially 150 ~ 200 bp in length, the library fragments were purified with the AMPure XP system (Beckman Coulter, Beverly, USA). Then 3 µL USER Enzyme (NEB, USA) was used with size-selected, adaptor-ligated cDNA at 37 °C for 15 min followed by 5 min at 95 °C before PCR. Then PCR was performed with Phusion High-Fidelity DNA polymerase, universal PCR primers and index (X) primers. Finally, PCR products were purified (AMPure XP system) and library quality assessed on the Agilent Bioanalyzer 2100 system. The clustering of the index-coded samples was performed on a cBot Cluster Generation System using the TruSeq PE Cluster Kit v3-cBot-HS (Illumina). After cluster generation, eighteen libraries preparations were sequenced on the Illumina NovaSeq 6000 and 150 bp paired-end reads were generated. Reads containing more than 10 % poly-N and more than 50 % low-quality reads (Q ≤ 20) were removed from the raw data using Trimmomatic v0.33 [36]. Concurrently, the Q20 and Q30 values, GC content, and sequence duplication level of the clean data were calculated. All downstream analyses were based on clean, high quality data. Clean reads were then mapped against the reference genome using Hisat2 [14].

### Identification of Differential Expressed Genes (DEGs) and transcription factors

In order to quantify the gene expression, the count-based method, FeatureCounts [37] was used. Finally, the transcript counts were used for pairwise differential gene expression analysis using the DEseq2 package [21]. A cut-off value of log2 ratio ± 1 and P-value 0.05 were used to filter out the significant transcripts in each case. Further, transcription factors were identified in *L. chinensis*. Transcription factor prediction was carried out by iTAK [15] software and HMMSCAN [16] was used to identify TFs. Transcription factors were then compared to plant specific transcription factor database PlnTFDB [17].

### Functional annotation and classification

Gene Ontology (GO, http://www.geneontology.org/) enrichment analysis of the DEGs was implemented using GOseq (v. 1.22) [38] using Wallenius’noncentral hypergeometric distribution, which can adjust for gene length bias in DEGs. Affected pathways were determined using Kyoto Encyclopedia of Genes and Genomes (KEGG, http://www.kegg.jp) [39]. We used KOBAS [40] software to annotate and identify the enriched KEGG pathways of the DEGs.

### Selection of candidate non-target genes

Genes that were commonly expressed between treated S and T, and non-treated S and T, and between treated T and treated S plants were selected for further evaluation based on their gene ontologies (GO). Genes that were assigned with GO molecular function and biological process related to metabolism and signaling pathways (oxidoreductase activity, nuclear acid binding transcription factor activity, hydrolase activity, transferase activity, transmembrane transporter activity, transferase activity, protein transporter activity, biosynthetic process, small molecule metabolic process, signal transduction, homeostatic process, immune system process, cell wall organization, secondary metabolic process, nitrogen cycle metabolic process) were evaluated based on UniProt and their foldchange. Genes with predicted annotations related to stress response, signaling, transcription factors, and herbicide metabolism were selected as potential candidate NTSR genes involved in cyhalofop-butyl tolerance.

### Phylogenetic analysis

To build a neighbor-joining tree, we selected amino acid sequences of candidate genes and obtained amino acid sequences of homologous genes in *A. thaliana*, rice, sorghum, maize, and soybean from the NCBI (https://www.ncbi.nlm.nih.gov/) and CYP databases (https://drnelson.uthsc.edu/CytochromeP450.html), as well as amino acid sequences of identified genes involved in herbicide metabolic pathways. We constructed a phylogenetic tree using MEGAX [41] with 1000 bootstrap replicates.

### qRT-PCR validation of RNA-Seq expression patterns

Primers to amplify candidate genes were designed based on sequence of the identified genes and listed in Additional file 12. The 18 s rRNA and Actin were used as internal control genes in qRT-PCR.

Total RNA was extracted and purified using the TransGen RNA extraction kit (TranGen, Beijing, China). 1 µg of RNA was used for first-stand cDNA synthesis using the FastQuant RT Kit (Tiangen, Beijing, China). qRTPCR was conducted in 96-well plates on the ABI 7500 real time PCR system (ABI Life Technologies) using SuperReal PreMix Plus (SYBR Green) (Tiangen, Beijing,China). Reactions were conducted in a 20 µL volume with three replicates for each cDNA sample. Each reaction mixture included 10 µL 2 × SuperReal PreMix Plus, 1 µL diluted cDNA, 0.6 µL primers, 0.4 µL 50 × ROX reference dye, and 7.4 µL RNase-free ddH2O. qRT-PCR programs consisted of 15 min incubation at 95 °C, 40 cycles of 95 °C for 10 s, 56 °C for 20 s and 72 °C for 32 s. At the end of the amplification cycle, a melting analysis was carried out to verify the absence of non-specific amplification. Similar amplification efficacy of the target and internal control genes (86.0-99.8 %) were observed. Fold-change in gene expression (as 2-△Ct) was calculated by the comparative CT method [42], relative to the susceptible samples, where △CT = [CT target geneCT mean of two internal control genes]. 

## Supplementary Information

**Additional file 1.** Standardized ID, experimental design and quality control information for each sample.

**Additional file 2. **Readcounts of each gene in samples.

**Additional file 3. **The differentially expressed gene list between TC and SC.

**Additional file 4. **The differentially expressed gene list between TE and SE.

**Additional file 5. **The differentially expressed gene list between SE and SC.

**Additional file 6. **The differentially expressed gene list between TE and TC.

**Additional file 7. **A) The volcano plot of differentially expressed genes in SE versus SC. B) Gene ontology (GO) analysis of induced-DEGs in SE versus SC. The DEGs were summarized in biological process, cellular component and molecular function. C) KEGG annotation of induced-DEGs in SE versus SC. The Rich factor is the ratio of the number of DEGs annotated in a pathway term to the total number of genes in that pathway.

**Additional file 8. **A) The volcano plot of differentially expressed genes in TE versus TC. B) Gene ontology (GO) analysis of induced-DEGs in TE versus TC. The DEGs were summarized in biological process, cellular component and molecular function. C) KEGG annotation of induced-DEGs in TE versus TC. The Rich factor is the ratio of the number of DEGs annotated in a pathway term to the total number of genes in that pathway.

**Additional file 9. **Table of transcription factors predicted in the whole transcriptome.

**Additional file 10. **Information on candidate genes involved in regulating cyhalofop-butyl resistance of LC18002.

**Additional file 11. ** The results of multiple sequence alignment with protein sequences of all cytochrome P450 family genes in *L. chinensis* and some cytochrome P450 family genes that have been identified in other species.

**Additional file 12. **Primer information for qRT-PCR validation.

**Additional file 13. ** Protein sequences which were used to construct phylogenetic tree.

**Additional file 14. **The Gene list of Venn diagram.

## Data Availability

The datasets supporting the conclusions of this article are included within the article and its additional files. The raw Illumina sequence reads have been deposited in the NCBI Sequence Read Archive (SRA) database with accession number PRJNA697925.

## Abbreviations

ACCase: Acetyl-CoA carboxylase
ALS: Acetolactate synthase
BP: Biologic process
CC: Cellular component
DEGs: Differentially expressed genes
TPM: Transcripts per million reads
GO: Gene ontology
GST: Glutathione S-transferase
KEGG: Kyoto encyclopedia of genes and genomes
MF: Molecular function
NTSR: Non-target-site based resistance
TSR: Target-site based resistance
RNA-Seq: High-through output RNA-sequencing
TF: Transcript factor
