# Supplementary material for: Whole transcriptome analysis resulted in the identification of Chinese sprangletop (Leptochloa chinensis) genes involved in cyhalofop-butyl tolerance
Source: BMC Genomics. 2021 Jul 9;22:521. doi: 10.1186/s12864-021-07856-z (PMC8268407; doi:10.1186/s12864-021-07856-z)
Supplement: Supplementary file 7 — Additional file 7. A) The volcano plot of differentially expressed genes in SE versus SC. B) Gene ontology (GO) analysis of induced-DEGs in SE versus SC. The DEGs were summarized in biological process, cellular component and molecular function. C) KEGG annotation of induced-DEGs in SE versus SC. The Rich factor is the ratio of the number of DEGs annotated in a pathway term to the total number of genes in that pathway. [file 12864_2021_7856_MOESM7_ESM.pdf]

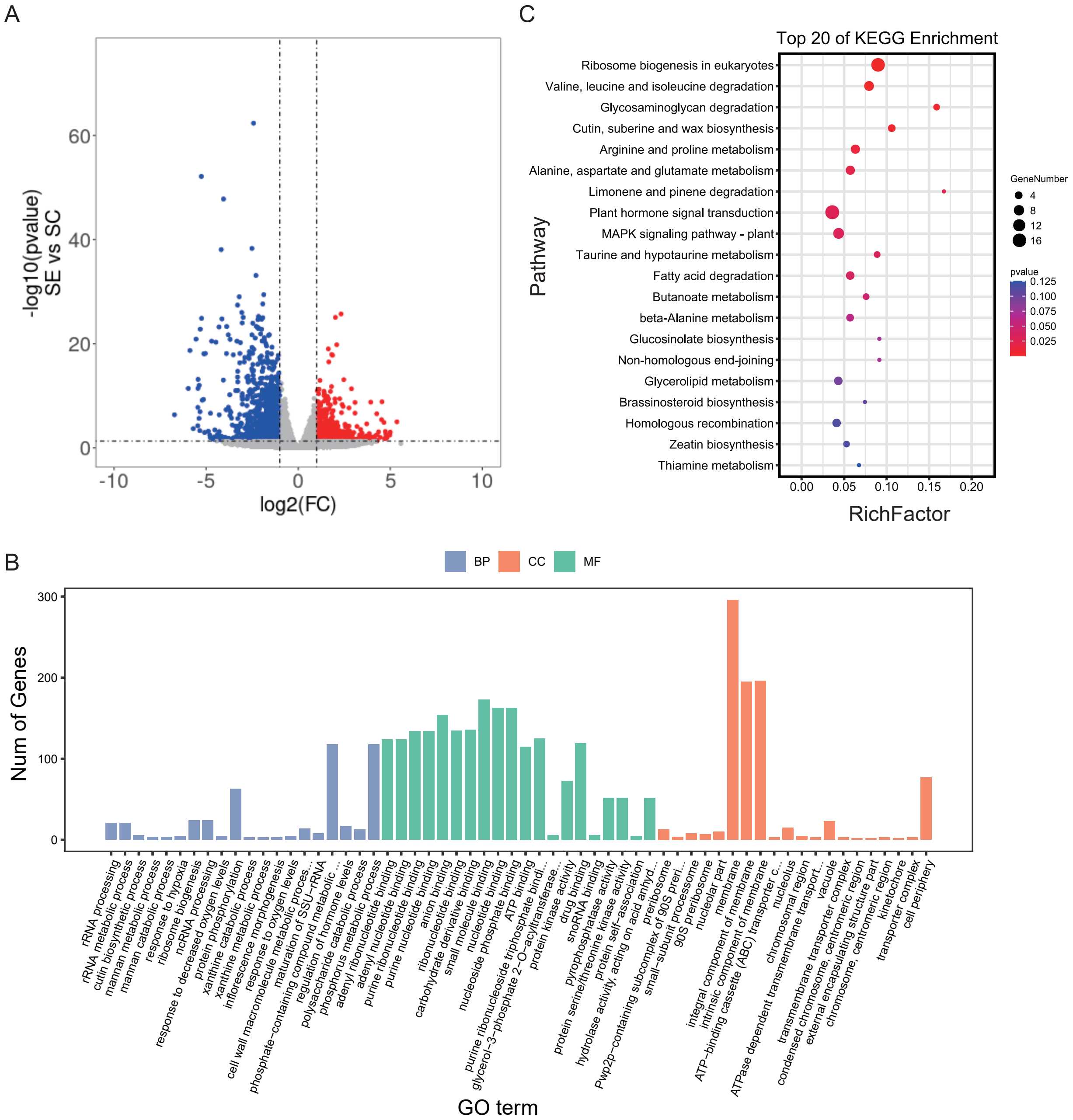
A
-log10(pvalue)
SE vs SC
log2(FC)
-10
-5
0
5
10
0
20
40
60
C
Top 20 of KEGG Enrichment
Pathway
RichFactor
Ribosome biogenesis in eukaryotes
Valine, leucine and isoleucine degradation
Glycosaminoglycan degradation
Cutin, suberine and wax biosynthesis
Arginine and proline metabolism
Alanine, aspartate and glutamate metabolism
Limonene and pinene degradation
Plant hormone signal transduction
MAPK signaling pathway - plant
Taurine and hypotaurine metabolism
Fatty acid degradation
Butanoate metabolism
beta-Alanine metabolism
Glucosinolate biosynthesis
Non-homologous end-joining
Glycerolipid metabolism
Brassinosteroid biosynthesis
Homologous recombination
Zeatin biosynthesis
Thiamine metabolism
0.00
0.05
0.10
0.15
0.20
GeneNumber
4
8
12
16
pvalue
0.125
0.100
0.075
0.050
0.025
B
BP
CC
MF
Num of Genes
GO term
0
100
200
300
rRNA processing
rRNA metabolic process
cutin biosynthetic process
mannan metabolic process
mannan catabolic process
response to hypoxia
ribosome biogenesis
ncRNA processing
response to decreased oxygen levels
protein phosphorylation
xanthine catabolic process
xanthine metabolic process
inflorescence morphogenesis
response to oxygen levels
cell wall macromolecule metabolic proces...
maturation of SSU−rRNA
phosphate−containing compound metabolic ...
regulation of hormone levels
polysaccharide catabolic process
phosphorus metabolic process
adenyl ribonucleotide binding
adenyl nucleotide binding
purine ribonucleotide binding
purine nucleotide binding
anion binding
ribonucleotide binding
carbohydrate derivative binding
small molecule binding
nucleotide binding
nucleoside phosphate binding
ATP binding
purine ribonucleoside triphosphate bindi...
glycerol−3−phosphate 2−O−acyltransferase...
protein kinase activity
drug binding
snoRNA binding
pyrophosphatase activity
protein serine/threonine kinase activity
protein self−association
hydrolase activity, acting on acid anhyd...
preribosome
Pwp2p−containing subcomplex of 90S preri...
small−subunit processome
90S preribosome
nucleolar part
membrane
integral component of membrane
intrinsic component of membrane
ATP−binding cassette (ABC) transporter c...
nucleolus
chromosomal region
ATPase dependent transmembrane transport...
vacuole
transmembrane transporter complex
condensed chromosome, centromeric region
external encapsulating structure part
chromosome, centromeric region
kinetochore
transporter complex
cell periphery
