# Supplementary figures and images for: Whole transcriptome analysis resulted in the identification of Chinese sprangletop (Leptochloa chinensis) genes involved in cyhalofop-butyl tolerance
Source: BMC Genomics. 2021 Jul 9;22:521. doi: 10.1186/s12864-021-07856-z (PMC8268407; doi:10.1186/s12864-021-07856-z)

A

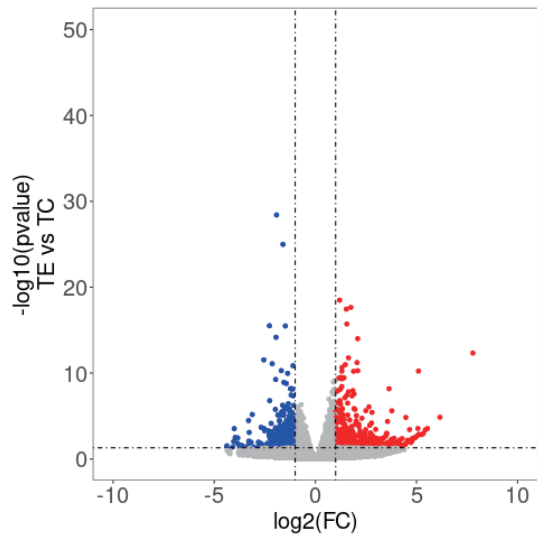

B

BP CC MF

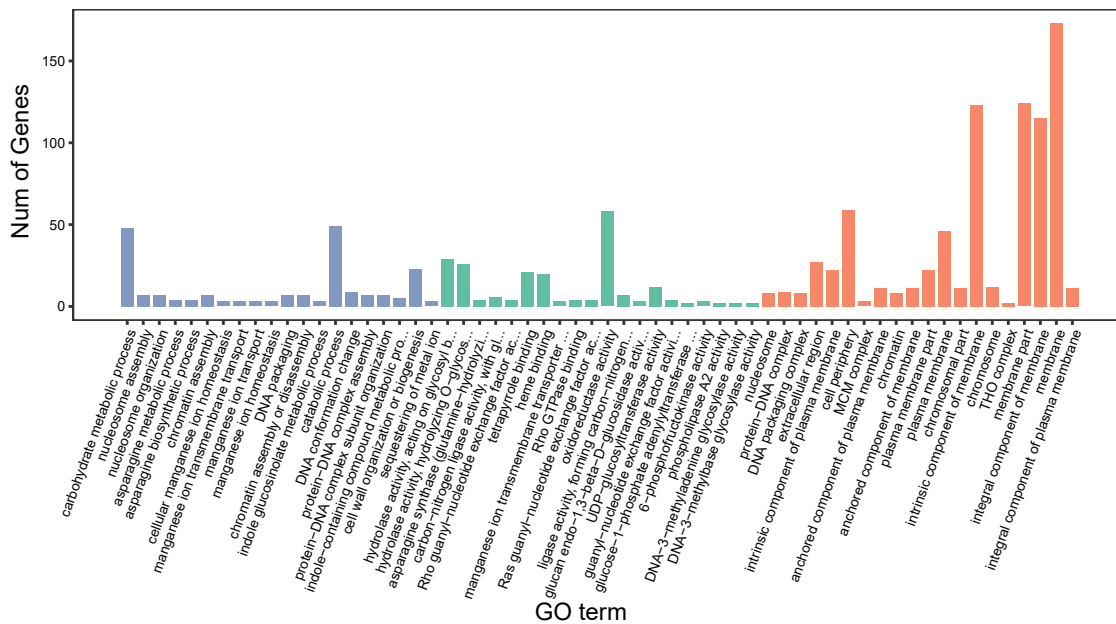

C

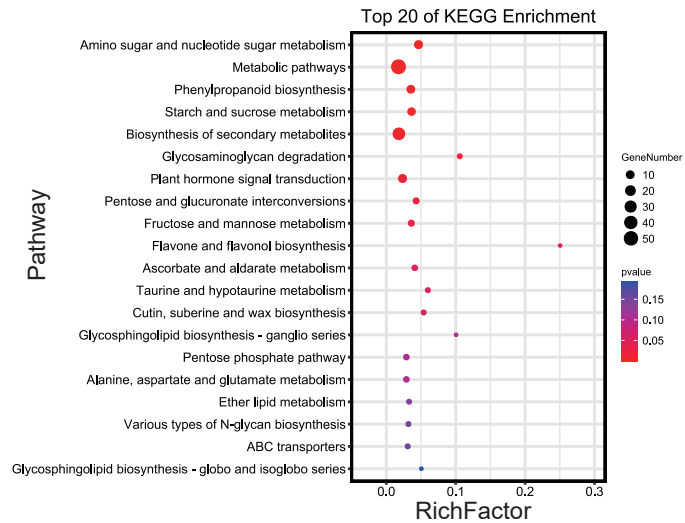

Supplement: Supplementary file 8 — Additional file 8. A) The volcano plot of differentially expressed genes in TE versus TC. B) Gene ontology (GO) analysis of induced-DEGs in TE versus TC. The DEGs were summarized in biological process, cellular component and molecular function. C) KEGG annotation of induced-DEGs in TE versus TC. The Rich factor is the ratio of the number of DEGs annotated in a pathway term to the total number of genes in that pathway. [file 12864_2021_7856_MOESM8_ESM.pdf]
